# Combined Effects of Lanthanum (III) and Acid Rain on Antioxidant Enzyme System in Soybean Roots

**DOI:** 10.1371/journal.pone.0134546

**Published:** 2015-07-31

**Authors:** Xuanbo Zhang, Yuping Du, Lihong Wang, Qing Zhou, Xiaohua Huang, Zhaoguo Sun

**Affiliations:** 1 State Key Laboratory of Food Science and Technology, Jiangsu Coorperative Innovation Center of Water Treatment Technology and Materials, Environment and Civil Engineering, Jiangnan University, Wuxi, Jiangsu, China; 2 Jiangsu Collaborative Innovation Center of Biomedical Functional Materials, Jiangsu Key Laboratory of Biomedical Materials, College of Chemistry and Materials Science, Nanjing Normal University, Nanjing, Jiangsu, China; National Taiwan University, TAIWAN

## Abstract

Rare earth element pollution (REEs) and acid rain (AR) pollution simultaneously occur in many regions, which resulted in a new environmental issue, the combined pollution of REEs and AR. The effects of the combined pollution on the antioxidant enzyme system of plant roots have not been reported. Here, the combined effects of lanthanum ion (La^3+^), one type of REE, and AR on the antioxidant enzyme system of soybean roots were investigated. In the combined treatment of La^3+^ (0.08 mM) and AR, the cell membrane permeability and the peroxidation of cell membrane lipid of soybean roots increased, and the superoxide dismutase, catalase, peroxidase and reduced ascorbic acid served as scavengers of reactive oxygen species. In other combined treatments of La^3+^ (0.40 mM, 1.20 mM) and AR, the membrane permeability, malonyldialdehyde content, superoxide dismutase activity, peroxidase activity and reduced ascorbic acid content increased, while the catalase activity decreased. The increased superoxide dismutase activity, peroxidase activity and reduced ascorbic acid content were inadequate to scavenge the excess hydrogen peroxide and superoxide, leading to the damage of the cell membrane, which was aggravated with the increase in the concentration of La^3+^ and the level of AR. The deleterious effects of the combined treatment of La^3+^ and AR were stronger than those of the single treatment of La^3+^ or AR. Moreover, the activity of antioxidant enzyme system in the combined treatment group was affected directly and indirectly by mineral element content in soybean plants.

## Introduction

Rare earth elements (REEs) exhibit useful physical and chemical properties that enable their wide applications in petroleum, metallurgy, textiles, ceramics, glassmaking, new materials (catalyst, permanent magnet, optical and hydrogen-storage material) production, and medicines [1–3]. At the suitable concentrations, REEs are also used in agriculture to improve the yield and quality of crops [4]. The uses of REEs have accelerated the accumulation of REEs in soils [5], which has become a global environmental issue [6–7]. For example, the average contents of REEs in soils in China, Australia, Japan and German are 197.67, 104.30, 97.57 and 15.48 mg/kg, respectively [7]. The accumulation of REEs in soils inevitably affects plant growth [8–11]. Antioxidant enzyme system in plants is the important protective mechanism in the response to stress [9]. It has been reported that REEs at suitable concentrations could promote plants to resist environmental stress (e.g. acid rain, heavy metals, ozone, low temperature, salinity, drought, and so on) by increasing the antioxidant capacity of plants [12–14]. However, little information on the concern on the potential risks of high-concentration REEs on the antioxidant enzyme system of plants has been presented [15–16].

Acid rain (AR) is a global environmental issue [17]. When its pH level reaches a certain damage threshold, AR inhibits the growth of plants through direct deposition to leaves as well as indirect acidification of surface water and soil [17]. It subsequently changes the plant population structure and finally inhibits the community functions [17–18]. It has been reported that AR exerts deleterious effects on both the physiological and biochemical characteristics of various plants [19–21]. The studies on cucumber (*Cucumis sativus* L.), muskmelon (*Cucumis melo* L.) and birch (*Betula pendula* R.) indicated that the effects of AR on the activities of superoxide dismutase (SOD), catalase (CAT) and peroxidase (POD) depend on the plant species, the pH level of AR and the duration of treatments [22–24]. The simultaneous pollution of REEs and AR occurs in many regions[25], and it produces a new environmental issue, i.e. the combined pollution of REEs and AR. Thus, it is very important to investigate the combined effects of REEs and AR on plants, including the combined effects of low-concentration REEs (or the improvement concentration) and AR on plants, which is commonly ignored; and the combined effects of REEs and AR at the current and the future levels. The action mechanism of the combined pollution of La^3+^ and AR on plants remains largely unclear although we have tried to clarify the mechanism from the view of photosynthesis in leaves [26]. Plant roots are the important compartments of the combined pollution of REEs and AR, and they absorb nutrients and moisture from soil. Therefore, the investigations on the combined effects of REEs and acid rain on plant roots are of great significance. Our group has previously conducted a preliminary study on the effects of the combined pollution of La^3+^ and AR on root phenotype of soybean [27]. In order to further understand the response mechanism, the studies on the combined effects of REEs and AR on antioxidant enzyme system in plant roots are important, and these effects have not been reported.

Lanthanum (La), the first lanthanide element in periodic table, is ubiquitous in soils [5]. Soybean is an important economic crop and recommended for the study on phytotoxicity by the US EPA. Thus, in this study, the combined effects of La^3+^ and AR on antioxidant enzyme system, cell membrane and metal element contents in soybean roots were investigated. The results could provide some references for the scientifically evaluating the potential ecological risk of REEs and AR on plants.

## Materials and Methods

### Preparation of Solutions

The control rain at pH 7.0 was prepared by adding Ca^2+^, Na^+^, K^+^, NH_4_^+^, Mg^2+^, SO_4_^2-^, NO_3_^-^, F^-^ and Cl^-^ to deionised water, in which the Ca^2+^, Na^+^, K^+^, NH_4_^+^, Mg^2+^, SO_4_^2-^, NO_3_^-^, F^-^ and Cl^-^ contents were 0.83 μM, 1.32 μM, 0.15 μM, 5.34 μM, 0.36 μM, 0.64 μM, 0.47 μM, 0.69 μM and 1.80 μM, respectively. The ionic composition was derived from precipitation data in the southeast of China [28–29]. The simulated AR at pH 3.0, 3.5 and 4.5 were prepared by adjusting the pH of control rain with the additions of the concentrated H_2_SO_4_ and HNO_3_ in a ratio of 1.10:1 (v/v, by chemical equivalents) [28–29].

The-P nutrient solution was prepared by replacing 1 mM KH_2_PO_4_ in the Hoagland’s solution (pH 7.0) with 1 mM KCl to avoid precipitation of lanthanum phosphate.

The La^3+^ solutions with different concentrations (0.08, 0.40 or 1.20 mM) were prepared by dissolving appropriate quantities of LaCl_3_ (Sigma-Aldrich, USA) in-P nutrient solution.

### Plant Culture and Treatment

Soybeans were cultured as described in our previous study [8, 27]. The soybean seeds (Zhonghuang 25, Wuxi Seed Co., Ltd., China) were sterilized in a HgCl_2_ (0.1%) solution for 5 min, rinsed several times with distilled water and germinated in an incubator at 25 ± 1°C. Three uniform seedlings with a radicle length of approximately 0.5 cm were transplanted into each pot (diameter = 15 cm, height = 30 cm) containing the 1.0 kg air-dried substrate (vermiculite and pearlite, 1:1, v/v). The-P nutrient solution was added to maintain substrate water content of 60%. Pots were placed in a greenhouse at 25 ± 3°C, a day/night cycle of 16/8 h, and a relative humidity of 70 − 80%. Photosynthetic photon flux density provided by the incandescent lamps at the greenhouse was selected as 300 μmol m^-2^ s^-1^, based on the fact that the light saturation point of soybean plant is usually 250~500 μmol^-2^ s^-1^ [30–31] and the light saturation point of soybean plant treated with La^3+^ and AR was approximately 310 μmol^-2^ s^-1^, measured with photometer (Fluke 941, US). The-P nutrient solution was used to irrigate the plants and to maintain substrate water content of 60%, and 1 mM KH_2_PO_4_ was sprayed on the leaves every other day to apply the required inorganic phosphate of plants.

Filling-stage soybean plants were subjected to 16 treatments of La^3+^ and AR in four classes [27], e.g. the control treatment, single treatment of La^3+^ or AR as well as the combined treatment of La^3+^ and AR. First was the control treatment. Soybean plants were irrigated with the-P nutrient solution (pH 7.0) and sprayed with the control rain. The substrate water content was 60%. Second was the La^3+^ treatment. Soybean plants were irrigated with the La^3+^ solution (0.08, 0.40 or 1.20 mM, pH 7.0) and then sprayed with the control rain. The substrate water content was 60%. Third was the AR treatment. Soybean plants were irrigated with the-P nutrient solution and sprayed with AR (pH 3.0, 3.5, 4.5). The substrate water content was 60%. Fourth was the combined treatment of La^3+^ and AR. Soybeans were irrigated with the La^3+^ solution (0.08, 0.40 or 1.20 mM, pH 7.0), and then sprayed with AR. The water content of substrate was 60%. Soybeans were sprayed with AR by a sprayer. The diameter of drop was approximately 0.5 mm, which caused the optimal detention time and distribution area of AR on the surface of leaves. For the 16 treatments of La^3+^ and AR, the amount of simulated AR or control rain was 300 mL per pots, which was calculated according to the precipitation and evaporation in the southeast of China. All treatments were replicated in five pots, and KH_2_PO_4_ solution was sprayed every other day to apply the required inorganic phosphate of plants. After the La^3+^ and AR treatment for 7 d, the roots were collected for the determination of the test indices.

### Determination of Membrane Permeability

Membrane permeability measurements were based upon the previous method [32]. Leaves were sliced to yield three 3×9 mm discs, representing the central pith, midparenchyma and cortex of the tuber tissue. The twenty four discs were rinsed, placed into 40 mL deionized water and gently tumbled at ambient room temperature. Conductance of deionized water was measured after 15 min (*C*_1_), 2 h (*C*_2_), and 2 h after a freeze-thaw treatment (*C*_total_). The membrane permeability was expressed as %/h = 100 × (*C*_2_-*C*_1_)/(1.75 *C*_total_).

### Determination of Malonyldialdehyde (MDA)

The level of lipid peroxidation was expressed as the content of MDA [33]. Samples (0.5 g) were repeatedly extracted with the mixed solution of ethanol and water (4:1, v/v) containing 1 mg L^-1^ butylated hydroxytoluene (BHT) using the sonication. After centrifugation, supernatants were pooled and an aliquot of appropriately diluted sample was added to a test tube with an equal volume of either (1)–thiobarbituric acid (TBA) solution containing 20% (w/v) trichloroacetic acid and 0.01% (w/v) BHT, or (2) +TBA solution containing the above materials plus 0.65% TBA. Samples were heated at 95°C for 25 min and after cooling, the absorbance was read at 440 nm, 532 nm, and 600 nm, respectively. The content of MDA was expressed as equation: MDA (nmol/mL) = 10^6^ × (*A*-*B*)/157000; *A* = *Abs*_532+TBA_-*Abs*_600+TBA_- (*Abs*_532-TBA_-*Abs*_600-TBA_); *B* = (*Abs*_440+TBA_-*Abs*_600+TBA_) × 0.0571. Here 157000 was the molar extinction coefficient for MDA. The molar absorbance of 1–10 mM sucrose at 532 nm and 440 nm was 8.4 and 147, respectively, giving a ratio of 0.0571.

### Determination of Hydrogen Peroxide (H_2_O_2_) Content

Hydrogen peroxide content was determined by the previous method with some modifications [34]. Root tissues (0.5 g) were homogenized in an ice bath with 3% (w/v) trichloroacetic acid. The homogenate was centrifuged at 12,000×g for 15 min, and 1 mL of supernatant was added to 1 mL of 100 mM potassium phosphate buffer (pH 7.0) and 2 mL of 1 M KI. The absorbance was measured at 390 nm. The content of H_2_O_2_ was calculated based on a standard curve.

### Determination of Superoxide (O_2_^-^) Content

Superoxide content was determined by a modified method according to Elstner and Heupel [35]. Two grams of root tissue were homogenized in 3 mL of 3% trichloride acetic acid. The homogenate was centrifuged at 12,000×g for 15 min, and 1 mL of supernatant was added to 1 mL of 50 mM potassium phosphate buffer (pH 7.0) containing 1 mM hydroxylammonium chloride and the mixture was incubated in 25°C for 20 min. The mixture was then incubated with 2 mL of 17 mM sulfanilic acid and 2 mL of 7 mM α-naphthyl amine at 25°C for 20 min. The final solution was mixed with an equal volume of ether, and the absorbance of the pink phase was measured at 530 nm. The content of O_2_^-^ was calculated based on a standard curve.

### Determination of Superoxide Dismutase (SOD), Catalase (CAT) and Peroxidase (POD) Activities

Roots (5 g) were homogenized in 50 mM potassium phosphate buffer (pH 7.8) including 5 mM ascorbic acid, 5 mM dithiothreitol, 5 mM ethylene diamine tetraacetic acid (EDTA), and 2% (v/v) polyvinylpyrrolidone. The homogenates were centrifuged at 15,000×g for 15 min and the supernatants were used for enzyme activity assaying.

The SOD activity was determined essentially as described by Spychalla and Desborough [36]. Each 3 mL of reaction mixture contained 50 mM Na_2_CO_3_/NaHCO_3_ buffer, pH 10.2, 0.1 mM EDTA, 0.015 mM ferricytochrome c, and 0.05 mM xanthine. One unit of SOD was defined as the amount of enzyme that which caused 50% inhibition of the rate of ferricytochrome c reduction.

The CAT activity was determined by following the consumption of H_2_O_2_ at 240 nm according to the literature [37]. Each 3 mL of reaction mixture contained 100 mM potassium phosphate buffer, pH 7.0, and 50 μL of the enzyme extract. The reaction was initiated by adding 15 mM H_2_O_2_.

The POD activity was determined by the literature [38]. The reaction mixture contained phosphate buffer (25 mM, pH 7.0), guaiacol (0.05%), H_2_O_2_ (10 mM), and crude peroxidase. Activity was determined by the increase per minute in the absorbance at 470 nm due to guaiacol oxidation (E = 26.6 mM cm^-1^).

### Determination of Reduced Ascorbic Acid (AsA) Content

The reduced AsA content was determined by high performance liquid chromatography (HPLC) (1100LC, Agilent, USA) at 254 nm according to previous research [39]. Plant tissues (1 g) were homogenized in liquid nitrogen and extracted in ice-cold metaphosphoric acid 5% (w/v). The extract was centrifuged for 10 min at 6,000 × g. The column employed was the Alltima C18 column (4.6 × 250 mm, 5 μm; Alltech Italia srl). The mobile phase consisted of 0.05 M sodium acetate and acetonitrile (95:5, v:v, pH 2.8). Isocratic elution was selected. The temperature of the column was adjusted at 26°C and the flow rate at 1 mL min^-1^. The total run time was 15 min. Calibration was achieved using purified ascorbic acid as standard.

### Determination of Mineral Element and La Contents

The mineral element and La contents in soybean roots and leaves were determined by inductively coupled plasma mass spectrometry (ICP-MS) (POEMS, Thermo Jarrel Ash, USA) [40–41]. The roots and leaves were collected, cleaned and washed three times with deionized water. These roots were dried in an oven and crushed into 1 mm segments. Then 0.5 g samples were digested with 8 mL oxidizing solution (15 M HNO_3_ and 9 M H_2_O_2_, v/v) for 30 min at 2600 kPa (80 psi) in a MDS-2000 microwave oven (CEM Corp., Matthews, NC, USA). The samples were diluted with deionized water to a final volume of 25 mL for determination of La and mineral element contents. In addition, standard solutions were used for the calibration.

### Statistical Analysis

Each treatment was replicated five times. All values were presented as the means ±SD. The significance of differences between different treatments was analyzed by one-way ANOVA using SPSS 17. The interaction between La^3+^ and AR was analyzed by two-way ANOVA.

## Results

### Combined Effects of La^3+^ and AR on Membrane Permeability and MDA Content of Soybean Roots

Table 1 shows the effects of La^3+^ and AR on membrane permeability and MDA content of soybean roots. When soybean roots were treated with 0.08 mM La^3+^, the membrane permeability and MDA content of soybean roots were unchanged compared with those of the control (Table 1). When the concentration of La^3+^ increased to 0.40 (1.20) mM, the membrane permeability and MDA content of roots increased by 49.27% (58.19%) and 21.18% (40.51%), respectively, in comparison with the control (Table 1).

**Table 1:** Effects of La^3+^ and AR on membrane permeability, MDA content, H_2_O_2_ and O_2_^-^ content in soybean roots.

AR (pH)	La^3+^ (mM)	Membrane permeability (%)	MDA content (nmol·g^-1^)	H_2_O_2_ content (μmol·g^-1^)	O_2_^-^ content (μg·g^-1^)
**3.0**	**0.00**	46.90±1.07^a^h^b^ (133.09)	18.20±0.51f(125.17)	1.04±0.02f(120.00)	2.38±0.02g(129.70)
	**0.08**	52.60±0.99fg(149.27)	19.32±0.51e(132.87)	1.13±0.01e(130.41)	2.53±0.02f(137.60)
	**0.40**	64.32±1.04c(182.54)	23.00±0.53b(158.18)	1.34±0.02b(154.49)	2.89±0.04c(157.43)
	**1.20**	74.21±1.06a(210.60)	25.78±0.59a(177.30)	1.42±0.02a(163.25)	3.11±0.03a(169.26)
**3.5**	**0.00**	45.45±0.55h(128.99)	17.27±0.86fg(118.78)	0.98±0.02g(113.13)	2.25±0.03h(122.45)
	**0.08**	50.70±0.68g(143.88)	18.05±0.56f(124.14)	1.07±0.02f(123.62)	2.36±0.01g(128.77)
	**0.40**	63.23±0.51d(179.43)	21.60±0.69c(148.56)	1.29±0.02c(148.16)	2.78±0.04d(151.33)
	**1.20**	69.07±1.26b(196.02)	25.01±0.69a(172.01)	1.35±0.02b(155.92)	3.01±0.02b(164.14)
**4.5**	**0.00**	35.64±2.72j(101.14)	14.00±0.58h(96.29)	0.89±0.03i(101.74)	1.80±0.02k(98.22)
	**0.08**	42.80±0.53i(121.46)	16.40±0.42g(112.79)	0.97±0.01gh(111.18)	2.13±0.02i(115.80)
	**0.40**	53.70±0.83f(152.39)	19.75±0.60de(135.83)	1.21±0.02d(138.94)	2.67±0.03e(145.23)
	**1.20**	62.30±0.75d(176.80)	22.38±0.31bc(153.92)	1.28±0.02c(147.00)	2.85±0.02c(155.14)
**7.0**	**0.00**	35.24±1.27j(100.00)	14.54±0.53h(100.00)	0.87±0.01i(100.00)	1.84±0.03j(100.00)
	**0.08**	36.00±1.60j(102.16)	14.94±0.59h(102.75)	0.95±0.02h(109.45)	1.89±0.05j(102.94)
	**0.40**	52.60±1.32fg(149.27)	17.62±0.68f(121.18)	1.15±0.02e(132.60)	2.24±0.02h(121.52)
	**1.20**	55.74±1.00e(158.19)	20.43±0.73d(140.51)	1.22±0.02d(140.32)	2.41±0.04fg(130.92)
***F***		7.034	2.701	8.630	12.614
***p***		*	*	*	*

^a^ Values are means ± standard deviation errors, *n* = 5.

^b^ Significantly differences at *p* < 0.05 were showed with different letter in each column.

* Significance at 0.05 levels.

The treatment of AR at pH 4.5 did not change the membrane permeability and MDA content of soybean roots. When the pH value of AR decreased to 3.5, the membrane permeability and MDA content increased by 28.99% and 18.78%, respectively, compared with those of the control (Table 1), and the higher increase in the membrane permeability and MDA content in the treatment of AR at pH 3.0 (33.09% and 25.17%) were observed.

When soybean roots were treated with 0.08 mM La^3+^ and AR at pH 4.5, the membrane permeability was increased by 21.46%, 19.30% and 20.32%, respectively, compared with those of the control and the single treatment of 0.08 mM La^3+^ or AR at pH 4.5 (Table 1). Similarly, the increase degrees in the MDA content were as follows: 12.79%, 10.04% and 16.50%. Relative to the control treatment and the single treatment of La^3+^ or AR, the membrane permeability and MDA content in other combined treatments increased. The increase degrees rose with the increase in the La^3+^ concentration and the decrease in the pH value of AR (Table 1). The results of two-way ANOVA indicated that there was an obvious interaction between La^3+^ and AR that affect the membrane permeability and MDA content in soybean roots (Table 1). The increase degrees of membrane permeability and MDA content caused by the combined treatment were less than the sum of those caused by the single treatment of La^3+^ or AR, and that is namely synergistic effect.

### Combined Effects of La^3+^ and AR on H_2_O_2_ and O_2_^-^ Contents of Soybean Roots

When soybean roots were treated with 0.08 mM La^3+^, the H_2_O_2_ content of roots increased by 9.45%, while the O_2_^-^ content was unchanged, compared with that of the control. When the concentration of La^3+^ was increased to 0.40 and 1.20 mM, the H_2_O_2_ and O_2_^-^ contents of roots were still higher than those of the control (Table 1).

When soybean roots were treated with AR at pH 4.5, the H_2_O_2_ and O_2_^-^ contents of roots were unchanged, compared with those of the control. For the treatment of AR at pH 3.5 or 3.0, in comparison with the control, the H_2_O_2_ and O_2_^-^ contents increased, and the increase extents were as followed: 13.13% or 20.00% for H_2_O_2_ content, and 22.45% or 29.70% for O_2_^-^ content, respectively (Table 1). When soybean roots were treated with 0.08 mM La^3+^ and AR at pH 4.5, the H_2_O_2_ content of roots increased by 11.18%, 1.73% and 9.44%, respectively, compared with that of the control and the single treatment of 0.08 mM La^3+^ or AR at pH 4.5 (Table 1). Similarly, the extents of increase in O_2_^-^ content were as follows: 15.80%, 12.86% and 17.58%. The similar effects were observed in other combined treatment groups. The increased effects rose as the level of La^3+^ and AR increased (Table 1). The results of two-way ANOVA indicated that there was an obvious interaction between La^3+^ and AR that affects H_2_O_2_ content and O_2_^-^ content in soybean roots and the interaction is synergistic effect (Table 1). The membrane permeability (MDA content) was positively correlated with the H_2_O_2_ and O_2_^-^ contents (*p*<0.05) (Table 2).

**Table 2:** Linear regression equation and correlation coefficient between the membrane permeability, MDA content and the H_2_O_2_ and O_2_^-^ contents in soybean roots treated with La^3+^ and AR.

Membrane permeability		MDA content	
Linear regression equation	Correlation coefficient (*R*)	Linear regression equation	Correlation coefficient (*R*)
y_1_ = 66.003x_1_-21.666	0.967**	y_2_ = 19.552x_1_-2.712	0.901**
y_1_ = 27.815x_2_-15.526	0.932**	y_2_ = 8.446x_2_-1.397	0.944**

y_1_, y_2_ represent the membrane permeability and MDA content, respectively. x_1_, x_2_ represent the H_2_O_2_ content and O_2_^-^ content, respectively.

** Significance at 0.01 levels.

### Combined Effects of La^3+^ and AR on the SOD, CAT and POD Activities as well as the Reduced AsA Content in Soybean Roots

Table 3 shows the effects of La^3+^ and AR on the SOD, CAT and POD activities as well as the reduced AsA content in soybean roots. When soybean roots were treated with 0.08 mM La^3+^, the SOD and CAT activities in soybean roots increased by 6.04% and 15.81%, respectively, while the POD activity and the reduced AsA content were unchanged, compared with the control. As the concentration of La^3+^ increased to 0.40 and 1.20 mM, the CAT activity was lower, and the SOD activity, POD activity and reduced AsA content significantly were higher than those of the control (Table 3).

**Table 3:** Effects of La^3+^ and AR on the SOD, CAT and POD activities and reduced AsA content in soybean roots.

AR (pH)	La^3+^ (mM)	SOD activity (U·g_FW_^-1^)	CAT activity [mgH_2_O_2_·(min·g)^-1^]	POD activity [Δ470·(g.min)^-1^]	Reduced AsA content (μg·g^-1^)
**3.0**	**0.00**	717.01±21.53^a^g^b^(134.44)	1.50±0.03c(127.95)	84.0±2.61h(119.49)	53.19±1.14f(118.94)
	**0.08**	756.99±33.45f(141.94)	1.76±0.01a(150.44)	89.6±2.52g(127.45)	56.70±1.14ef(126.84)
	**0.40**	1066.67±51.45b(200.00)	0.85±0.03j(72.74)	105.1±2.07bc(149.50)	69.72±2.26ab(155.94)
	**1.20**	1183.66±52.44a(221.94)	0.74±0.02k(62.91)	111.6±1.39a(158.75)	71.89±1.69a(160.80)
**3.5**	**0.00**	636.99±21.53h(119.44)	1.34±0.02e(114.70)	77.7±1.39i(110.53)	49.42±1.42g(110.55)
	**0.08**	697.54±51.45g(130.79)	1.65±0.03b(140.98)	85.1±0.98h(121.05)	55.37±1.39f(123.86)
	**0.40**	929.03±35.96cd(174.19)	0.96±0.03hi(82.05)	100±1.57de(142.25)	62.21±1.64cd(139.13)
	**1.20**	964.93±50.12c(180.92)	0.81±0.03j(69.32)	107.6±0.57b(153.06)	67.41±0.95b(150.77)
**4.5**	**0.00**	542.90±20.60ij(101.79)	1.20±0.02f(102.68)	70.2±1.80j(99.85)	44.40±1.33i(99.32)
	**0.08**	623.01±26.21h(116.81)	1.41±0.02d(120.92)	71.6±2.10j(101.85)	53.73±0.47f(120.19)
	**0.40**	832.69±35.80e(156.13)	1.04±0.03gh(89.06)	96.1±0.89f(136.70)	59.79±1.79de(133.75)
	**1.20**	890.97±38.53d(167.06)	0.93±0.03i(79.57)	102±2.43cd(145.09)	64.05±1.52c(143.28)
**7.0**	**0.00**	533.33±18.35i(100.00)	1.17±0.01f(100.00)	70.3±3.05j(100.00)	44.71±0.31h(100.00)
	**0.08**	565.57±18.28j(106.04)	1.35±0.06e(115.81)	71.1±3.08j(101.14)	46.59±0.96h(104.2)
	**0.40**	609.68±28.11h(114.31)	1.09±0.04g(93.16)	87.1±2.26gh(123.85)	57.20±0.78e(127.95)
	**1.20**	791.83±36.36ef(148.47)	0.99±0.03h(84.70)	98.3±2.12ef(139.83)	61.41±2.01d(137.37)
***F***		10.169	52.863	3.889	2.228
***p***		*	*	*	*

^a^ Values are means ± standard deviation errors, *n* = 5.

^b^ Significantly differences at *p* < 0.05 were showed with different letter in each column.

* Significance at 0.05 levels.

In comparison of the control, the SOD, CAT and POD activity and reduced AsA content were unchanged in roots treated with AR at pH 4.5. When the pH value of AR decreased to 3.5 and 3.0, the SOD, CAT and POD activities as well as the reduced AsA content were significantly increased compared with those of the control (Table 3).

When soybean roots were treated with 0.08 mM La^3+^ and AR at pH 4.5, the SOD and CAT activities as well as the reduced AsA content increased by 16.81%, 20.92% and 20.19%, respectively, while the activity of POD was unchanged, compared with those of the control (Table 3). In other combined treatments of La^3+^ and AR, the SOD and POD activities as well as the reduced AsA content were significantly increased, the activity of CAT was decreased compared with those of the control (Table 3). The results of two-way ANOVA indicated that there was an obvious interaction between La^3+^ and AR that affected the SOD, CAT and POD activities, as well as the reduced AsA content in soybean roots (Table 3). The H_2_O_2_ content (O_2_^-^ content) negatively correlated with the CAT activity, and positively correlated with the SOD activity, POD activity and reduced AsA content (*p*<0.05) (Table 4).

**Table 4:** Relationship between the H_2_O_2_ (O_2_^-^) contents and the CAT activity, POD activity and reduced AsA content in soybean roots treated with La^3+^ and AR.

H_2_O_2_ content		O_2_^-^ content	
Linear regression equation	Correlation coefficient (*R*)	Linear regression equation	Correlation coefficient (*R*)
y_1_ = 0.001x_1_+0.500	0.816	y_2_ = 0.002x_1_+0.911	0.880
y_1_ = -0.378x_2_+1.574	-0.671**	y_2_ = -0.796x_2_+3.392	-0.595**
y_1_ = 0.012x_3_+0.053	0.895**	y_2_ = 0.028x_3_+0.028	0.915**
y_1_ = 0.020x_4_-0.008	0.896**	y_2_ = 0.046x_4_+0.188	0.864**

y_1_, y_2_ represent the H_2_O_2_ content and O_2_^-^ content, respectively. x_1_, x_2_, x_3_ and x_4_ represent the SOD activity, CAT activity, POD activity and reduced AsA content, respectively.

** Significance at 0.01 levels.

### Combined Effects of La^3+^ and AR on the Contents of Mineral Elements in Soybean

Table 5 showed the contents of macroelements (K, Ca and Mg) and microelements (Cu, Mn, Zn and Fe) in soybean roots and leaves. When soybean roots were treated with 0.08 mM La^3+^, the contents of K, Ca, Mg and Mn in the roots and leaves were decreased, compared with those of the control. This effect was more evident at higher concentration of La^3+^ (0.40 and 1.20 mM), excepted for Mg in leaves. The Cu, Fe and Zn contents in the roots and leaves treated with La^3+^ were increased compared with those of the control except that the Zn content in the leaves decreased at 1.20 mM of La^3+^. The increased effects rose as the level of La^3+^ increased. When soybean roots were treated with AR, the K and Mg contents in the roots and leaves were increased, the Ca content in these organs was unchanged, the Cu, Mn, Fe and Zn contents in the roots and leaves (excepted for Zn content in the roots) were increased, compared with those of the control.

**Table 5:** Effects of La^3+^ and AR on the contents of mineral elements and La in soybean.

AR (pH)	La^3+^ (mM)	K (%)	Ca (%)	Mg (%)	Fe (μg g^-1^)	Mn (μg g^-1^)	Cu (μg g^-1^)	Zn (μg g^-1^)	La (μg g^-1^)
**Roots**									
**3.0**	**0.00**	2.39±0.06^a^bc^b^ (105.78)	0.58±0.01a (110.90)	1.34±0.03b (106.50)	334.08±6.33h 146.64)	69.65±1.52b (111.18)	9.00±0.15e (126.36)	37.03±0.77i (75.29)	0.18±0.00h (135.06)
	**0.08**	1.96±0.03d (86.60)	0.53±0.01c (100.33)	0.99±0.02de (78.35)	370.30±9.19f (162.54)	36.04±0.85f (57.53)	10.7±0.17c (150.18)	55.44±0.91f (112.73)	57.46±1.05f (42390.96)
	**0.40**	1.55±0.03h (68.42)	0.34±0.01f (65.13)	0.95±0.02ef (75.27)	516.19±9.65c (226.58)	26.43±0.58h (42.19)	11.01±0.18bc (154.48)	42.55±0.8h (86.52)	209.45±4.14c (154532.39)
	**1.20**	1.22±0.02i (53.80)	0.27±0.01i (52.63)	0.8±0.01g (63.34)	655.27±12.19a (287.63)	21.24±0.39ij (33.91)	7.41±0.12hi (103.94)	31.79±0.73j (64.64)	222.41±6.68a (164094.42)
**3.5**	**0.00**	2.59±0.05a (114.28)	0.56±0.01ab (107.89)	1.37±0.03b (108.97)	249.55±4.21ij (109.54)	101.81±2.78a (162.52)	9.22±0.18e (129.36)	41.23±0.75h (83.83)	0.18±0.00h (133.978)
	**0.08**	2.41±0.06b (106.56)	0.55±0.01abc (105.67)	1.01±0.02d (79.86)	365.47±9.31fg (160.42)	38.52±0.64f (61.5)	11.86±0.19a (166.39)	57.69±0.96ef (117.31)	56.63±1.30f (41780.06)
	**0.40**	1.77±0.03e (78.5)	0.39±0.01e (74.92)	0.82±0.02g (65.23)	450.00±9.62d (197.53)	23.84±0.54hi (38.06)	11.33±0.20b (158.98)	42.07±0.86h (85.54)	175.36±4.58d (129378.92)
	**1.20**	1.32±0.02i (58.37)	0.29±0.01gh (56.54)	0.57±0.01h (44.99)	570.75±11.57b (250.53)	18.09±0.36k (28.88)	10.83±0.17c (151.99)	32.11±0.56j (65.29)	211.25±6.20bc (155860.45)
**4.5**	**0.00**	2.63±0.15a (116.43)	0.55±0.01abc (105.84)	1.57±0.03a (124.58)	251.97±5.23ij (110.60)	67.39±1.12b (107.58)	8.39±0.15f (117.81)	43.52±0.76h (88.5)	0.11±0.00h (81.58)
	**0.08**	2.42±0.04b (107.14)	0.46±0.01d (89.10)	0.91±0.02f (72.46)	341.32±6.98gh (149.82)	52.61±1.04e (83.978)	11.07±0.18bc (155.36)	49.16±1.24g (99.96)	42.26±0.84g (31182.13)
	**0.40**	1.73±0.03ef (76.51)	0.44±0.01d (83.96)	0.49±0.02i (38.70)	459.66±10.93d (201.77)	20.07±0.48jk (32.039)	10.11±0.18d (141.95)	62.16±1.18d (126.40)	183.2±3.68d (135169.27)
	**1.20**	1.59±0.04fh (70.56)	0.30±0.01g (58.14)	0.34±0.01j (27.35)	505.54±8.97c (221.91)	18.03±0.48k (28.78)	9.74±0.15d (136.66)	74.12±1.32b (150.72)	214.13±4.82ab (157985.34)
**7.0**	**0.00**	2.26±0.05c (100.00)	0.52±0.01c (100.00)	1.26±0.03c (100.00)	227.82±4.88j (100.00)	62.64±1.42c (100.00)	7.13±0.15i (100.00)	49.18±0.90g (100.00)	0.146±0.00h (100.00)
	**0.08**	2.36±0.05bc (104.61)	0.55±0.01ab (106.17)	0.9±0.02f (71.47)	259.21±6.17i (113.78)	58.02±1.2d (92.63)	7.92±0.13g (111.13)	60.04±1.00de (122.09)	40.82±0.81g (30116.73)
	**0.40**	1.56±0.03h (68.88)	0.54±0.01bc (102.88)	0.51±0.02i (40.48)	413.77±9.32e (181.63)	29.57±0.69g (47.20)	10.11±0.19d (141.84)	68.07±1.22c (138.42)	144.97±2.83e (106961.26)
	**1.20**	1.33±0.03i (58.76)	0.35±0.01f (67.19)	0.29±0.01k (22.69)	464.49±10.49d (203.89)	31.18±0.75g (49.77)	7.64±0.13gh (107.20)	84.17±1.45a (171.15)	203.08±4.78c (149831.05)
***F***		86.455	112.614	394.345	201.875	477.839	84.197	230.695	698.207
***p***		*	*	*	*	*	*	*	*
**Leaves**									
**3.0**	**0.00**	3.17±0.05b (124.06)	2.98±0.07ab (98.64)	1.27±0.03a (131.46)	273.70±4.67c (141.08)	35.31±0.80d (121.23)	7.64±0.12bc (141.98)	55.14±1.58ab (119.43)	0.28±0.0j (142.43)
	**0.08**	2.02±0.03f (78.99)	2.78±0.06def (92.06)	1.16±0.02b (120.10)	289.18±5.44b (149.06)	24.96±0.94hi (85.71)	8.08±0.14a (150.21)	49.19±0.85def (106.55)	1.29±0.02fg (646.57)
	**0.40**	1.87±0.03f (73.04)	2.88±0.07ad (95.18)	0.97±0.02d (100.24)	280.91±4.66bc (144.80)	22.00±0.74j (75.52)	6.92±0.11e (128.53)	45.36±1.04h (98.25)	1.19±0.03g (597.77)
	**1.20**	1.37±0.02g (53.60)	2.31±0.04h (76.26)	1.03±0.02c (106.89)	308.5±5.05a (159.01)	21.39±0.82j (73.44)	4.96±0.01h (92.11)	55.98±1.20ab (121.25)	1.45±0.03e (726.36)
**3.5**	**0.00**	3.40±0.07a (132.99)	2.99±0.06ab (98.79)	0.98±0.02cd (101.73)	268.76±5.14c (138.53)	46.34±0.97b (159.10)	7.96±0.15ab (147.87)	57.05±1.08a (123.58)	0.30±0.01j (151.82)
	**0.08**	2.75±0.06c (107.50)	2.81±0.06cde (93.05)	0.9±0.02e (93.13)	256.80±4.34d (132.37)	27.21±0.93fgh (93.42)	7.41±0.24cd (137.79)	53.89±1.21bc (116.74)	0.87±0.02i (438.73)
	**0.40**	1.46±0.03g (57.01)	2.65±0.05fg (87.45)	0.82±0.01f (85.20)	290.61±5.05b (149.79)	27.89±0.84fg (95.75)	7.02±0.11de (130.45)	46.2±0.98fgh (100.07)	2.02±0.05d (1014.39)
	**1.20**	0.90±0.01h (35.32)	2.67±0.06efg (88.30)	0.88±0.01e (91.04)	305.15±5.18a (157.26)	29.21±0.58f (100.30)	5.753±0.09g (106.79)	53.69±1.13bc (116.30)	4.01±0.08a (2014.81)
**4.5**	**0.00**	2.74±0.04c (107.40)	2.96±0.06abc (97.94)	0.96±0.02d (99.28)	227.82±4.50fg (117.43)	50.81±1.07a (174.45)	6.73±0.22ef (125.10)	51.51±1.03cde (111.57)	0.24±0.00j (119.52)
	**0.08**	2.31±0.04e (90.24)	2.84±0.05bd (93.83)	0.89±0.02e (92.50)	238.50±4.59ef (122.93)	32.63±0.73e (112.05)	7.80±0.19abc (144.99)	45.56±1.10 (98.69)	1.08±0.02h (539.75)
	**0.40**	0.91±0.02h (35.59)	2.83±0.05bd (93.76)	0.85±0.01ef (87.71)	256.80±4.28d (132.37)	33.74±0.83de (115.83)	8.24±0.14a (153.16)	48.62±1.05efg (105.31)	1.38±0.03ef (690.85)
	**1.20**	0.47±0.01i (18.23)	2.85±0.06bd (94.18)	0.98±0.02cd (101.71)	249.55±3.96de (128.63)	38.34±0.62c (131.62)	7.9±0.13ab (146.91)	49.12±0.85defg (106.4)	2.61±0.05c (1309.54)
**7.0**	**0.00**	2.55±0.19d (100.00)	3.02±0.06a (100.00)	0.97±0.02d (100.00)	194.01±3.68i (100.00)	29.12±1.39f (100.00)	5.38±0.13gh (100.00)	46.17±1.02gh (100.00)	0.20±0.00j (100.00)
	**0.08**	2.64±0.05cd (103.48)	2.96±0.06abc (98.01)	0.87±0.02ef (89.37)	210.91±3.90h (108.71)	25.83±0.53gh (88.68)	6.41±0.17f (119.07)	51.71±0.97cd (112.01)	0.90±0.02i (450.83)
	**0.40**	0.82±0.01h (31.99)	2.80±0.05def (92.51)	0.87±0.02ef (90.47)	218.16±3.60gh (112.45)	22.74±0.84ij (78.07)	6.59±0.22ef (122.58)	47.21±0.98fgh (102.26)	1.47±0.04e (736.22)
	**1.20**	0.41±0.01i (16.17)	2.55±0.04g (84.33)	0.86±0.02ef (88.98)	225.40±4.89g (116.18)	22.69±0.89ij (77.91)	6.94±0.14e (129.01)	39.78±0.65i (86.17)	2.96±0.05b (1484.68)
***F***		260.631	11.263	38.990	56.762	98.209	40.238	19.556	909.757
***p***		*	*	*	*	*	*	*	*

^a^ Values are means ± standard deviation errors, *n* = 5.

^b^ Significantly differences at *p* < 0.05 were showed with different letter in each column.

* Significance at 0.05 levels.

When soybean roots were treated with both 0.08 mM La^3+^ and acid rain at pH 4.5, the K, Ca, Mg and Mn contents were decreased, but the Cu, Zn and Fe contents were increased, compared with those of the control. These effects were more evident in other combined treatments of La^3+^ and AR. The results of two-way ANOVA indicated that there was an obvious interaction between La^3+^ and AR that affected the contents of mineral elements in the roots and leaves (Table 5). The K, Ca content in the roots and leaves, and Mn content in roots positively correlated with the CAT activity (excepted for Ca in leaves), and negatively correlated with the SOD activity, POD activity, reduced AsA content and La content in the roots (Table 6). The Fe content in the roots and leaves negatively correlated with the CAT activity (excepted for Fe in leaves), and positively correlated with the SOD activity, POD activity, reduced AsA content and La content in the roots (Table 6). The Mg content in the roots negatively correlated with the POD activity, reduced AsA content and La content (Table 6). The Cu content in the leaves positively correlated with the CAT activity (Table 6).

**Table 6:** Correlation coefficients between antioxidant system activities and metal contents in soybean treated with La^3+^ and AR.

	SOD activity	CAT activity	POD activity	Reduced AsA content	La content (leaves)	La content (roots)
**Roots**						
**K content**	-0.775**	0.742**	-0.892**	-0.873**	-0.776**	-0.942**
**Ca content**	-0.852**	0.805**	-0.847**	-0.866**	-0.760**	-0.890**
**Mg content**	-0.439	0.492	-0.632**	-0.636**	-0.801**	-0.822**
**Fe content**	0.924**	-0.688**	0.955**	0.975**	0.715**	0.925**
**Mn content**	-0.682**	0.529*	-0.786**	-0.794**	-0.714**	-0.873**
**Cu content**	0.184	0.207	0.219	0.303	0.215	0.166
**Zn content**	-0.274	0.083	-0.051	-0.079	0.223	0.190
**La content**	0.823**	-0.786**	0.914**	0.910**	0.781**	1.00
**Leaves**						
**K content**	-0.518*	0.659**	-0.710**	-0.683**	-0.813**	-0.884**
**Ca content**	-0.717**	0.496	-0.730**	-0.741**	-0.580*	-0.705**
**Mg content**	0.037	0.405	-0.045	-0.072	-0.351	-0.361
**Fe content**	0.780**	-0.194	0.700**	0.690**	0.345	0.417
**Mn content**	-0.416	0.181	-0.452	-0.517*	-0.343	-0.489
**Cu content**	-0.217	0.519*	-0.160	-0.151	-0.146	-0.198
**Zn content**	0.047	0.207	-0.054	-0.099	-0.266	-0.305
**La content**	0.544*	-0.545*	0.715**	0.682**	1.00	0.781**

* Significance at 0.05 levels.

** Significance at 0.01 levels.

### Combined Effects of La^3+^ and AR on La Content of Soybean

When soybean roots were treated with La^3+^, the La contents in soybean increased compared with those of the control (Table 5). In addition, the increase was more evident as the La^3+^ concentration increased. The La content in soybean plants treated with AR was unchanged compared with that in the control (Table 5). In the combined treatments with La^3+^ (0.08, 0.40 or 1.20 mM) and acid rain at pH 4.5 (3.5 or 3.0), the La contents in the roots and leaves were increased, compared with that in the control and La^3+^ or AR single treatment (excepted for La in leaves). The results of two-way ANOVA indicated that there was an obvious interaction between La^3+^ and AR that affected the La contents in soybean roots and leaves (Table 5). Moreover, the La content in the roots and leaves positively correlated with the SOD activity, POD activity and reduced AsA content, but negatively correlated with the CAT activity (Table 6).

## Discussion

Aerobic biological metabolism produces active oxygen species (ROS), including H_2_O_2_, O_2_^-^, and ·OH [42]. The balance between the generation and scavenging of ROS exists in normal cells, which would not damage plants [10]. Abiotic stress can cause the excess accumulation of ROS in plants [10]. Excess ROS can rapidly attack all types of biomolecules such as nucleic acid, proteins, lipids, and amino acids [14], triggers free-radical chain reaction, and then causes membrane lipid peroxidation, which is one of the primary consequences of oxidative damage [14]. The injury level can be reflected by the MDA content and membrane permeability [43–44]. In plants, SOD, CAT and POD are major antioxidant enzymes, which can effectively remove the excess ROS to protect the plant cells from the damage of abiotic stress [22, 45]. AsA is the most abundant and powerful antioxidant in plants, and it can also prevent or minimize the damage caused by ROS [46]. In the present work, the effects of La^3+^ and AR on the antioxidant enzyme system of plant roots were understood.

The treatment of 0.08 mM La^3+^ did not affect the membrane permeability and MDA content in roots. Thus the generation and the elimination of ROS in roots remained in dynamic equilibrium under this treatment (Table 1). The treatments of 0.40 and 1.20 mM La^3+^ inhibited the CAT activity, promoted the SOD and POD activities, and increased the content of reduced AsA. The effects on the antioxidant enzyme system led to the accumulation of ROS (H_2_O_2_ and O_2_^-^) in roots, which induced the membrane lipid peroxidation and the increase in the membrane permeability (Tables 1 and 2). The same effects have been observed in rice (*Oryza sativa*) treated with Ce^3+^ and wheat (*Triticum aestivum*) treated with La^3+^ [9, 11, 47]. The treatment of AR at pH 4.5 did not affect the test indices. As the pH value of AR deceased, the SOD, CAT and POD activities were activated, meanwhile the Reduced AsA content increased, then H_2_O_2_ and O_2_^-^ excessively accumulated and finally induced significant damage in soybean roots. The similar results were also observed in cucumber (*Cucumis sativus*) and tomato (*Lycopersicon esculentum*) [22, 48].

In contrast with the single treatment with La^3+^ and AR, the combined treatment of La^3+^ and AR showed the different effects on the antioxidant enzyme system, which depended on the concentration of La^3+^ and the pH of AR (Tables 1 and 3). In the combined treatment of 0.08 mM La^3+^ and AR, H_2_O_2_ and O_2_^-^ serve as signaling molecules to activate SOD, POD, CAT and reduced AsA in root cells [10]. But the activated SOD, CAT, POD and reduced AsA can not efficiently eliminate the ROS, leading to the excess accumulation of ROS in roots (Table 1). Excess ROS rapidly attacked unsaturated fatty acids in cell membrane, induced membrane lipid peroxidation and increased the membrane permeability (Table 1). In the combined treatments of La^3+^ (0.40, 1.20 mM) and AR, the CAT activity was inhibited, on the contrary, the SOD activity, POD activity and the reduced AsA content were increased (Table 2). The activated SOD and POD, and the increased reduced AsA could remove H_2_O_2_, but the ROS (·OH and O_2_^-^) still excessively accumulated in soybean roots (Table 1). The excess ROS oxidized the unsaturated fatty acids in the membrane lipid of root cells [49], leading to the peroxidation of cell membrane lipid (Table 1) [43–44], the damage of the cell membrane and the destruction of the selective permeability of the cell membrane (Table 1). The deleterious effects on the cell membrane aggravated the electrolyte leakage from the cytoplasm (the membrane permeability). Meanwhile, we analyzed the interaction between La^3+^ and AR on the antioxidant enzyme system in soybean roots, and found that there was a synergistic effect of La^3+^ and AR on antioxidant enzyme system, ROS accumulation and membrane lipid peroxidation in soybean roots. We speculated that AR treatment made soybean roots absorb more La^3+^, leading to a higher accumulation of La^3+^ in the roots in comparison with the single treatment of La^3+^ [27]. Meanwhile, La^3+^ treatment promoted the uptake of H^+^ by roots compared with the single treatment of AR [50]. Anyway, as a new combined pollutant, the combined toxic effects should be paid more attention to.

Our results of correlation analysis showed that the K, Ca, Mg, Fe and Mn contents in the roots (the K, Ca and Fe contents in the leaves) positively (or negatively) correlated with the activities of antioxidant enzyme system in the roots (Table 6). These results were consistent with previous reports [51–52]. What’s more, the K, Ca, Mg, Fe and Mn contents in roots (K and Ca content in leaves) positively (or negatively) correlated with La content in the roots (Table 6), which disturbed the effect of La on the activities of antioxidant enzyme system in the roots [53–54].

## Conclusion

The combined treatment of La^3+^ and AR increase the membrane permeability and the peroxidation of membrane lipid. The increases resulted from the excess accumulation of H_2_O_2_ and O_2_^-^ together with the changes in the activities of the antioxidant enzymes and the content of antioxidant. Moreover, the changes in the contents of mineral elements in soybean plants directly and indirectly affected the activities of the antioxidant enzyme system in roots. These effects mentioned above were higher than those of the single treatment of La^3+^ or AR. Thus more attention should be paid on the potential threat of the combined pollution of La^3+^ and AR. Furthermore, in this study, the experimental design excluded soil and the effects from xenobiotics in soil (e.g., microbial metabolism, sorption). Therefore, the obtained results need to be confirmed by extending the investigation in real and natural soil-plant systems.
